# Complete Genome Sequence of *Mesorhizobium ciceri* Strain CC1192, an Efficient Nitrogen-Fixing Microsymbiont of *Cicer arietinum*

**DOI:** 10.1128/genomeA.00516-16

**Published:** 2016-06-09

**Authors:** Timothy Haskett, Penghao Wang, Joshua Ramsay, Graham O’Hara, Wayne Reeve, John Howieson, Jason Terpolilli

**Affiliations:** a Centre for Rhizobium Studies, Murdoch University, Perth, Australia; b School of Biomedical Sciences and Curtin Health Innovation Research Institute, Curtin University, Perth, Australia

## Abstract

We report the complete genome sequence of *Mesorhizobium ciceri* strain CC1192, an efficient nitrogen-fixing microsymbiont of *Cicer arietinum* (chickpea). The genome consists of 6.94 Mb distributed between a single chromosome (6.29 Mb) and a plasmid (0.65 Mb).

## GENOME ANNOUNCEMENT

*Cicer arietinum* (chickpea), a globally important grain legume, forms a N_2_-fixing symbiosis with soil bacteria (rhizobia) in the genus *Mesorhizobium* (1) that can be harnessed in agriculture to enable *C. arietinum* to be grown without nitrogen fertilizer (2). When cultivated in soil lacking compatible rhizobia, *C. arietinum* can be inoculated with a symbiotically effective strain of *Mesorhizobium*. In Australia, *Mesorhizobium ciceri* strain CC1192 has been the commercial inoculant for this crop for several decades (3, 4). Although CC1192 has been the only *C. arietinum* inoculant used, there is evidence in the field that *C. arietinum* is nodulated by genetically distinct strains (5), suggesting that novel *C. arietinum*–nodulating rhizobia have evolved during the past 30 years. *Mesorhizobium* spp. can harbor symbiotic genes on mobile chromosomal regions called “symbiosis islands” or integrative and conjugative elements (ICEs) (6, 7). Thus, the complete genome sequence of CC1192 will enable the investigation of horizontal gene transfer of symbiosis genes from this strain to soil rhizobia.

Strain CC1192 was sourced from the Australian inoculant industry mother culture (http://www.dpi.nsw.gov.au/content/agriculture/resources/soils/australian-inoculants-research-group) and confirmed to fix N_2_ effectively with *C. arietinum*, using established methods (8). CC1192 genomic DNA was extracted from a tryptone-yeast-grown culture (9) using a phenol-chloroform method as previously described (10). Whole-genome sequencing was performed by Macrogen (South Korea), using both Pacific BioSciences (PacBio) single-molecule real-time sequencing and Illumina HiSeq 2500 technology. Post filter, PacBio sequencing generated 96,824 trimmed reads with a mean read length of 11,579 bp, with Illumina sequencing generating an additional 19,850,308 paired-end reads. Raw Illumina reads were analyzed using FastQC version 0.10.1 (http://www.bioinformatics.babraham.ac.uk/projects/fastqc), and adaptors were removed by comparison against a comprehensive in-house adaptor sequence library. PacBio subreads were assessed using in-house software, and reads were automatically error-corrected in the assembly process.

The filtered Illumina and PacBio reads were assembled *de novo* using the hybrid approach of SPAdes assembler version 3.6.2 (11), with the number of mismatches and short indels reduced by incurring SPAdes’s postprocessing module MismatchCorrector, utilizing the BWA tool (12). The assembly obtained was scaffolded using SSPACE version 3.0 (13) and annotated using the NCBI Prokaryotic Genome Annotation Pipeline (PGAP) (http://www.ncbi.nlm.nih.gov/genomes/static/Pipeline.html). The genome consists of 6,943,628 bp with an average GC content of 62.49%. There are 6,642 coding sequences distributed between a single circular chromosome of 6,295,397 bp and a single plasmid of 648,231 bp.

As is the case with *M. loti* R7A (14), genes potentially involved in nodulation (nod) and nitrogen fixation (*nif* and *fix*) appear to be located on a 419-kb symbiosis island integrated within the chromosome of CC1192 (base pairs 4,216,431 to 4,635,338). This region also harbors a type IV secretion system, conjugative relaxase, biotin and nicotinate biosynthetic clusters, proteins known to control excision and transfer of ICE*Ml*Sym^R7A^ (15–17) and is flanked by direct repeat sequences. These characteristics are consistent with CC1192 carrying a mobile ICE. However, unlike ICE*Ml*Sym^R7A^, the CC1192 putative ICE is located adjacent to one of five serine tRNA genes and not a phenylalanine tRNA gene. Work is underway to investigate whether CC1192 can transfer its symbiotic capacity to non–*C. arietinum*–nodulating *Mesorhizobium* spp.

### Nucleotide sequence accession numbers.

The nucleotide sequence of the complete genome of CC1192 has been deposited in GenBank under the accession numbers CP015062 (chromosome) and CP015063 (plasmid pMc1192).
